# 2,2-Dibromo-1-(4-hydr­oxy-3-methoxy­phen­yl)ethanone

**DOI:** 10.1107/S1600536809020650

**Published:** 2009-06-06

**Authors:** Xiao-Hui Yang, Yong-Hong Zhou, Xing Song

**Affiliations:** a National Engineering Laboratory for Biomass Chemical Utilization; Key and Open Laboratory of Forest Chemical Engineering SFA, Institute of Chemical Industry of Forest Products CAF, Nanjing 210042, People’s Republic of China

## Abstract

The mol­ecule of the title compound, C_9_H_8_Br_2_O_3_, is stabilized by an intra­molecular O—H⋯O inter­action. Inter­molecular C—H⋯O inter­actions connect mol­ecules into a two-dimensional array in the *bc* plane; connections between these are afforded by π–π stacking inter­actions [centroid–centroid distance 3.596 (5) Å].

## Related literature

For the beta-O-4 substructure in lignin, see: Cathala *et al.* (2003 ▶). For attempts to prepare well defined linear polymers with the β-O-4 structure and to develop new methods of utilizing lignins, see: Kishimoto *et al.* (2005 ▶).
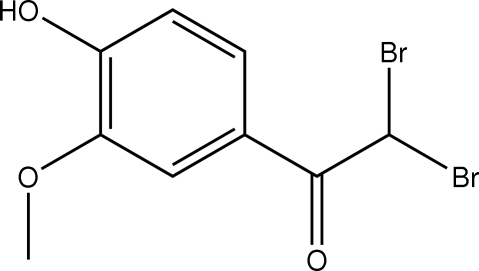


## Experimental

### 

#### Crystal data

C_9_H_8_Br_2_O_3_
*M* *_r_* = 323.97
Monoclinic, 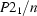

*a* = 7.0370 (14) Å
*b* = 10.805 (2) Å
*c* = 13.871 (3) Å
β = 98.80 (3)°
*V* = 1042.3 (4) Å^3^
*Z* = 4
Mo *K*α radiation
μ = 7.76 mm^−1^
*T* = 295 K
0.10 × 0.05 × 0.05 mm

#### Data collection

Enraf–Nonius CAD-4 diffractometer
Absorption correction: ψ scan (North *et al.*, 1968 ▶) *T*_min_ = 0.511, *T*_max_ = 0.698
2060 measured reflections
1900 independent reflections
894 reflections with *I* > 2σ(*I*)
*R*_int_ = 0.041
3 standard reflections every 200 reflections intensity decay: 1%

#### Refinement

*R*[*F* ^2^ > 2σ(*F* ^2^)] = 0.067
*wR*(*F* ^2^) = 0.159
*S* = 0.96
1900 reflections
127 parameters
61 restraints
H-atom parameters constrained
Δρ_max_ = 0.56 e Å^−3^
Δρ_min_ = −0.65 e Å^−3^

### 

Data collection: *CAD-4 EXPRESS* (Enraf–Nonius, 1994 ▶); cell refinement: *CAD-4 EXPRESS*; data reduction: *XCAD4* (Harms & Wocadlo, 1995 ▶); program(s) used to solve structure: *SHELXS97* (Sheldrick, 2008 ▶); program(s) used to refine structure: *SHELXL97* (Sheldrick, 2008 ▶); molecular graphics: *SHELXTL* (Sheldrick, 2008 ▶); software used to prepare material for publication: *SHELXTL*.

## Supplementary Material

Crystal structure: contains datablocks qj0709, I. DOI: 10.1107/S1600536809020650/tk2463sup1.cif

Structure factors: contains datablocks I. DOI: 10.1107/S1600536809020650/tk2463Isup2.hkl

Additional supplementary materials:  crystallographic information; 3D view; checkCIF report

## Figures and Tables

**Table 1:** Hydrogen-bond geometry (Å, °)

*D*—H⋯*A*	*D*—H	H⋯*A*	*D*⋯*A*	*D*—H⋯*A*
O2—H2*A*⋯O1	0.85	2.27	2.617 (11)	105
C1—H1*A*⋯O2^i^	0.96	2.51	3.398 (11)	153
C5—H5*A*⋯O3^ii^	0.93	2.57	3.460 (10)	161
C9—H9*A*⋯O3^ii^	0.98	2.38	3.222 (11)	143

Symmetry codes: (i) 
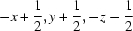
; (ii) 
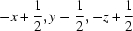
.

**Fig. 1.**
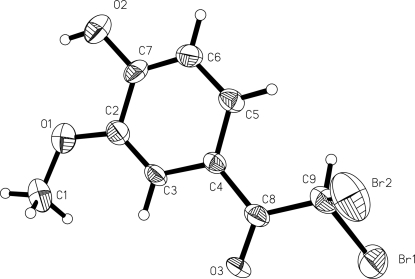
The molecular structure and atom-labeling scheme for (I), with displacement ellipsoids drawn at the 30% probability level.

## supplementary crystallographic information

### Comment

Lignin is natural polymer occurring in plant cell walls and is considered to be the second most abundant biopolymer after cellulose. The beta-O-4 structure is the most abundant substructure in lignin (Cathala *et al.*, 2003). In order to prepare well defined linear polymers composed of the β-O-4 structure and in attempt to develop new utilization methods of lignins (Kishimoto *et al.*, 2005), a new compound, 2,2-dibromo-1-(4-hydroxy-3-methoxyphenyl)ethanone, (I), was synthesized and its structure determined using single-crystal X-ray methods.

The molecular conformation of (I), Fig. 1, is stabilized by an intramolecular O—H···O interaction formed between the hydroxyl-H and methoxy-O atoms (H···O = 2.27 Å). The molecules are connected into a 2-D array via C-H···O interactions in the bc-plane (Table 1). Connections between the layers are afforded by π-π stacking interactions, with the shortest centroid···centroid distance being 3.596 (5)Å.

### Experimental

To a stirred solution of acetovanillone (5 g, 0.03 mol) in anhydrous CHCl_3_, bromine (3.1 ml, 0.06 mol) was added dropwise under nitrogen over 2 h at 273 K. The reaction mixture was kept at 273k for 1 h. The reaction mixture was diluted with ether and washed with ice-cold water and brine. The solution was dried over anhydrous Na_2_SO_4_ and concentrated to dryness *in vacuo*. The crude crystalline product was purified by column chromatography to obtain a pure white solid, (I). Colourless single crystals were grown by slow evaporation of an ethyl acetate solution of (I).

### Refinement

H atoms were placed in calculated positions and treated using a riding model, with C—H = 0.93–0.98 Å and O—H = 0.85 Å, and with *U*_iso_(H) = 1.2*U*_eq_(C, O) or 1.5*U*_eq_(C) for methyl-H atoms.

### Crystal data

**Table:**

C_9_H_8_Br_2_O_3_	*F*(000) = 624
*M**_r_* = 323.97	*D*_x_ = 2.065 Mg m^−^^3^
Monoclinic, *P*2_1_/*n*	Mo *K*α radiation, λ = 0.71073 Å
Hall symbol: -P 2yn	Cell parameters from 25 reflections
*a* = 7.0370 (14) Å	θ = 10–13°
*b* = 10.805 (2) Å	µ = 7.76 mm^−^^1^
*c* = 13.871 (3) Å	*T* = 295 K
β = 98.80 (3)°	Needle, colourless
*V* = 1042.3 (4) Å^3^	0.10 × 0.05 × 0.05 mm
*Z* = 4	

### Data collection

**Table:**

Enraf–Nonius CAD-4 diffractometer	894 reflections with *I* > 2σ(*I*)
Radiation source: fine-focus sealed tube	*R*_int_ = 0.041
graphite	θ_max_ = 25.3°, θ_min_ = 2.4°
ω/2θ scans	*h* = 0→8
Absorption correction: ψ scan (North *et al.*, 1968)	*k* = 0→12
*T*_min_ = 0.511, *T*_max_ = 0.698	*l* = −16→16
2060 measured reflections	3 standard reflections every 200 reflections
1900 independent reflections	intensity decay: 1%

### Refinement

**Table:**

Refinement on *F*^2^	Primary atom site location: structure-invariant direct methods
Least-squares matrix: full	Secondary atom site location: difference Fourier map
*R*[*F*^2^ > 2σ(*F*^2^)] = 0.067	Hydrogen site location: inferred from neighbouring sites
*wR*(*F*^2^) = 0.159	H-atom parameters constrained
*S* = 0.96	*w* = 1/[σ^2^(*F*_o_^2^) + (0.0723*P*)^2^] where *P* = (*F*_o_^2^ + 2*F*_c_^2^)/3
1900 reflections	(Δ/σ)_max_ < 0.001
127 parameters	Δρ_max_ = 0.56 e Å^−^^3^
61 restraints	Δρ_min_ = −0.65 e Å^−^^3^

### Special details

**Table:**

Geometry. All e.s.d.'s (except the e.s.d. in the dihedral angle between two l.s. planes) are estimated using the full covariance matrix. The cell e.s.d.'s are taken into account individually in the estimation of e.s.d.'s in distances, angles and torsion angles; correlations between e.s.d.'s in cell parameters are only used when they are defined by crystal symmetry. An approximate (isotropic) treatment of cell e.s.d.'s is used for estimating e.s.d.'s involving l.s. planes.
Refinement. Refinement of *F*^2^ against ALL reflections. The weighted *R*-factor *wR* and goodness of fit *S* are based on *F*^2^, conventional *R*-factors *R* are based on *F*, with *F* set to zero for negative *F*^2^. The threshold expression of *F*^2^ > σ(*F*^2^) is used only for calculating *R*-factors(gt) *etc*. and is not relevant to the choice of reflections for refinement. *R*-factors based on *F*^2^ are statistically about twice as large as those based on *F*, and *R*- factors based on ALL data will be even larger.

### Fractional atomic coordinates and isotropic or equivalent isotropic displacement parameters (Å^2^)

**Table:**

	*x*	*y*	*z*	*U*_iso_*/*U*_eq_	
Br1	0.08467 (19)	0.97920 (12)	0.38634 (8)	0.0775 (5)	
Br2	0.51183 (19)	0.91674 (13)	0.35768 (10)	0.0890 (5)	
O1	0.1990 (9)	1.1174 (7)	−0.1321 (4)	0.0521 (17)	
O2	0.2770 (9)	0.8866 (7)	−0.1677 (4)	0.062 (2)	
H2A	0.2526	0.9407	−0.2123	0.074*	
O3	0.2364 (10)	1.1382 (6)	0.2363 (4)	0.0578 (19)	
C1	0.1731 (15)	1.2472 (10)	−0.1180 (7)	0.065 (3)	
H1A	0.1408	1.2869	−0.1802	0.097*	
H1B	0.2900	1.2820	−0.0840	0.097*	
H1C	0.0712	1.2596	−0.0802	0.097*	
C2	0.2291 (13)	1.0450 (8)	−0.0514 (6)	0.041 (2)	
C3	0.2247 (12)	1.0754 (8)	0.0407 (5)	0.036 (2)	
H3A	0.2002	1.1572	0.0554	0.043*	
C4	0.2555 (12)	0.9894 (8)	0.1168 (5)	0.0303 (19)	
C5	0.2965 (12)	0.8669 (8)	0.0924 (5)	0.037 (2)	
H5A	0.3198	0.8071	0.1410	0.045*	
C6	0.3021 (13)	0.8348 (9)	−0.0047 (6)	0.043 (2)	
H6A	0.3279	0.7536	−0.0208	0.052*	
C7	0.2714 (13)	0.9187 (9)	−0.0728 (6)	0.044 (2)	
C8	0.2469 (13)	1.0318 (9)	0.2175 (6)	0.039 (2)	
C9	0.2485 (13)	0.9338 (9)	0.2920 (6)	0.048 (2)	
H9A	0.2046	0.8555	0.2606	0.057*	

### Atomic displacement parameters (Å^2^)

**Table:**

	*U*^11^	*U*^22^	*U*^33^	*U*^12^	*U*^13^	*U*^23^
Br1	0.0952 (10)	0.0694 (9)	0.0809 (7)	0.0149 (8)	0.0546 (7)	0.0129 (7)
Br2	0.0631 (8)	0.0828 (11)	0.1166 (10)	0.0081 (8)	−0.0004 (7)	0.0338 (8)
O1	0.051 (4)	0.059 (5)	0.046 (3)	−0.001 (4)	0.008 (3)	0.009 (3)
O2	0.065 (5)	0.072 (5)	0.055 (4)	0.001 (4)	0.030 (3)	−0.003 (4)
O3	0.105 (6)	0.018 (4)	0.058 (4)	0.001 (4)	0.037 (4)	−0.001 (3)
C1	0.072 (8)	0.055 (8)	0.068 (7)	−0.007 (7)	0.017 (6)	0.020 (6)
C2	0.044 (5)	0.035 (5)	0.045 (4)	−0.001 (4)	0.009 (4)	0.003 (4)
C3	0.041 (5)	0.020 (4)	0.048 (4)	−0.006 (4)	0.013 (4)	0.000 (3)
C4	0.027 (4)	0.024 (4)	0.040 (3)	−0.003 (4)	0.006 (3)	0.000 (3)
C5	0.038 (5)	0.031 (4)	0.041 (4)	0.004 (4)	−0.002 (4)	0.001 (4)
C6	0.046 (5)	0.034 (5)	0.052 (4)	0.000 (4)	0.015 (4)	−0.005 (4)
C7	0.046 (5)	0.048 (5)	0.045 (4)	0.002 (5)	0.025 (4)	−0.005 (4)
C8	0.042 (5)	0.025 (5)	0.053 (4)	0.004 (4)	0.019 (4)	0.001 (4)
C9	0.049 (5)	0.033 (5)	0.064 (5)	−0.002 (5)	0.015 (4)	0.004 (4)

### Geometric parameters (Å, °)

**Table:**

Br1—C9	1.935 (9)	C2—C7	1.437 (12)
Br2—C9	1.945 (9)	C3—C4	1.398 (10)
O1—C2	1.355 (10)	C3—H3A	0.9300
O1—C1	1.431 (12)	C4—C5	1.407 (11)
O2—C7	1.369 (9)	C4—C8	1.481 (11)
O2—H2A	0.8500	C5—C6	1.398 (11)
O3—C8	1.184 (10)	C5—H5A	0.9300
C1—H1A	0.9600	C6—C7	1.302 (11)
C1—H1B	0.9600	C6—H6A	0.9300
C1—H1C	0.9600	C8—C9	1.478 (12)
C2—C3	1.324 (11)	C9—H9A	0.9800
			
C2—O1—C1	117.4 (7)	C6—C5—H5A	119.9
C7—O2—H2A	119.6	C4—C5—H5A	119.9
O1—C1—H1A	109.5	C7—C6—C5	120.0 (9)
O1—C1—H1B	109.5	C7—C6—H6A	120.0
H1A—C1—H1B	109.5	C5—C6—H6A	120.0
O1—C1—H1C	109.5	C6—C7—O2	119.7 (9)
H1A—C1—H1C	109.5	C6—C7—C2	121.9 (8)
H1B—C1—H1C	109.5	O2—C7—C2	118.4 (8)
C3—C2—O1	129.0 (9)	O3—C8—C9	122.4 (8)
C3—C2—C7	118.0 (8)	O3—C8—C4	121.4 (8)
O1—C2—C7	112.9 (7)	C9—C8—C4	116.2 (8)
C2—C3—C4	122.7 (8)	C8—C9—Br1	110.5 (6)
C2—C3—H3A	118.7	C8—C9—Br2	107.5 (6)
C4—C3—H3A	118.7	Br1—C9—Br2	109.3 (4)
C3—C4—C5	117.2 (7)	C8—C9—H9A	109.8
C3—C4—C8	118.9 (7)	Br1—C9—H9A	109.8
C5—C4—C8	123.9 (7)	Br2—C9—H9A	109.8
C6—C5—C4	120.1 (8)		
			
C1—O1—C2—C3	5.5 (14)	O1—C2—C7—C6	−179.1 (8)
C1—O1—C2—C7	−174.4 (8)	C3—C2—C7—O2	−179.7 (9)
O1—C2—C3—C4	178.7 (8)	O1—C2—C7—O2	0.2 (12)
C7—C2—C3—C4	−1.3 (13)	C3—C4—C8—O3	−8.6 (13)
C2—C3—C4—C5	1.4 (13)	C5—C4—C8—O3	170.2 (9)
C2—C3—C4—C8	−179.7 (9)	C3—C4—C8—C9	170.2 (8)
C3—C4—C5—C6	−1.0 (12)	C5—C4—C8—C9	−10.9 (12)
C8—C4—C5—C6	−179.8 (8)	O3—C8—C9—Br1	35.2 (12)
C4—C5—C6—C7	0.7 (14)	C4—C8—C9—Br1	−143.6 (7)
C5—C6—C7—O2	−179.9 (8)	O3—C8—C9—Br2	−84.0 (10)
C5—C6—C7—C2	−0.7 (14)	C4—C8—C9—Br2	97.2 (8)
C3—C2—C7—C6	1.0 (14)		

### Hydrogen-bond geometry (Å, °)

**Table:**

*D*—H···*A*	*D*—H	H···*A*	*D*···*A*	*D*—H···*A*
O2—H2A···O1	0.85	2.27	2.617 (11)	105
C1—H1A···O2^i^	0.96	2.51	3.398 (11)	153
C5—H5A···O3^ii^	0.93	2.57	3.460 (10)	161
C9—H9A···O3^ii^	0.98	2.38	3.222 (11)	143

Symmetry codes: (i) −*x*+1/2, *y*+1/2, −*z*−1/2; (ii) −*x*+1/2, *y*−1/2, −*z*+1/2.
